# Thanks to all those who reviewed for *International Journal for Equity in Health* in 2015

**DOI:** 10.1186/s12939-016-0322-z

**Published:** 2016-03-07

**Authors:** Efrat Shadmi, Leiyu Shi

**Affiliations:** University of Haifa, Haifa, Israel; Johns Hopkins University, Baltimore, USA

## Abstract

The editors of *International Journal for Equity in Health* would like to thank all our reviewers who have contributed to the journal in Volume 14 (2015).

**Jens Aagaard-Hansen**

Denmark

**Muhammad Ahmed Abdullah**

Pakistan

**Jalelah Abdul-Raheem**

USA

**Aaron Abuosi**

Ghana

**Enoch Acheampong**

Ghana

**Olumide Adisa**

UK

**Filder Claret Adyero**

Uganda

**Dinesh Agarwal**

India

**Jun Aida**

UK

**Maryam Akbari**

Iran

**Sharareh Akhavan**

Sweden

**Shamima Akhter**

Bangladesh

**Patricia Akweongo**

Ghana

**Vassilis Aletras**

Greece

**Sara Allin**

Canada

**Alemayehu Ambel**

USA

**Djesika Amendah**

Kenya

**Sohail Amjad**

Pakistan

**Mary Amoakoh-Coleman**

Ghana

**Pernille Andersen**

Denmark

**Genevieve Aryeetey**

Ghana

**Likawunt Asfaw**

Ethiopia

**Gizachew Assefa Tessema**

Ethiopia

**Mona Backhans**

Sweden

**Juan Baeza**

UK

**David Baguma**

Uganda

**Ross Bailie**

Australia

**Petra Baji**

Hungary

**Muideen Bakare**

Nigeria

**Mate Balazs**

Hungary

**Enis Baris**

USA

**Emma Barnard**

Australia

**Murat Basaran**

Turkey

**Irmgard Bauer**

Australia

**Claudia Beiersmann**

Germany

**Cecilia Benoit**

Canada

**Anita Bercovitz**

USA

**Reshmi Bhageerathy**

India

**Jasmin Bhawra**

Canada

**Sibhatu Biadgilign**

Ethiopia

**Birpreet Birpreet**

Canada

**Ilse Blignault**

Australia

**Lennart Bogg**

Sweden

**Julia Bolívar Muñoz**

Spain

**Billie Bonevski**

Australia

**Margaret Boone**

Canada

**Elis Borde**

Brazil

**Lars Borgquist**

Sweden

**Kim Bosmans**

Belgium

**Abdesslam Boutayeb**

Morocco

**Mayer Brezis**

Israel

**Doerthe Brueggmann**

Germany

**Barbara Bucki**

France

**Sarah Burgard**

USA

**Alejandra Burgos**

Chile

**María Burrone**

Argentina

**Gerard Bury**

Ireland

**Luciana Butini Oliveira**

Brazil

**Silvia Calzón Fernández**

Spain

**Ines Campos-Matos**

Portugal

**Javier Campos-Serna**

Spain

**Lourdes Cantarero-Arevalo**

Denmark

**Gemma Carey**

Australia

**Marianne Carlsson**

Sweden

**Elaine Carnegie**

UK

**Miri Carter**

Switzerland

**Monica Castro**

Brazil

**Chien-Lung Chan**

Taiwan

**Shivani Chandra**

India

**Shu-Sen Chang**

Hong Kong

**Wei-Ching Chang**

Canada

**Thomas Charters**

Canada

**Keya Chatterjee**

India

**Brian Chen**

USA

**Mingsheng Chen**

China

**Wen Chen**

China

**Yingyao Chen**

China

**Zhuo Chen**

USA

**Jianquan Cheng**

UK

**Cecile Cherrier**

France

**Kyoung Hee Cho**

Korea, Republic of

**Eun-Kyong Choi**

USA

**Terkel Christiansen**

Denmark

**Roger Chung**

Hong Kong

**Songul Cinaroglu**

Turkey

**Ron Cisler**

USA

**Natalie Clark**

Canada

**Anne Cockcroft**

Botswana

**Anna-Britt Coe**

Sweden

**Claudia Coeli**

Brazil

**Donald Cole**

Canada

**Erica Cook**

UK

**Isabel Correia**

Portugal

**Jesus David Cortes Gil**

Mexico

**Bart Criel**

Belgium

**Jonathan Cylus**

UK

**Phyllis Dako-Gyeke**

Ghana

**Deborah De Moortel**

Belgium

**Jesús De Pedro-Cuesta**

Spain

**Anahit Demirchyan**

Armenia

**Niveditha Devasenapathy**

India

**Meenal Dhall**

India

**Angel Dilip**

Tanzania

**Coreen Domingo**

USA

**Antonia Domingo-Salvany**

Spain

**Javkhlanbayar Dorjdagva**

Mongolia

**Christopher Dowrick**

UK

**Zuzanna Drożdżak**

Switzerland

**Zhicheng Du**

China

**Esther Dungumaro**

Tanzania

**Jo Durham**

Australia

**Mark Dusheiko**

Switzerland

**Sue Dyson**

UK

**Andrew Edmonds**

USA

**Frida Eek**

Sweden

**Mary Egan**

Canada

**Bolaji Egbewale**

Nigeria

**François Eisinger**

France

**Fitriana Ekawati**

Indonesia

**Björn Ekman**

Sweden

**Syed Emdadul Haque**

Bangladesh

**Maria Emmelin**

Sweden

**Isabel Cristina Emmerick**

USA

**Jorge Escobedo**

Mexico

**Nilay Etiler**

Turkey

**Rebecca Evans**

Australia

**Alexandre Faisal-Cury**

Brazil

**Nicholas Fancourt**

USA

**Hai Fang**

China

**Wen-Hui Fang**

Taiwan

**Shingairai Feresu**

South Africa

**Astrid Fink**

Germany

**Kevin Fiscella**

USA

**Paul Fleming**

USA

**Steffen Flessa**

Germany

**Glenn Flores**

USA

**Elisabeth Fosse**

Norway

**Elizabeth Fradgley**

Australia

**Patricia Frenz**

USA

**Graciela Freyermuth**

Mexico

**Christine Friesen**

Canada

**John Furler**

Australia

**Jacqueline Gahagan**

Canada

**Charles Gallia**

USA

**Pooja Gangrade**

India

**Hongxia Gao**

China

**Manuel García-Goñi**

Spain

**Tony Gatrell**

UK

**Usha George**

Canada

**Claudia Geue**

UK

**Cristian Gherman**

Romania

**Luis Andres Gimeno-Feliu**

Spain

**Beatriz Gl Valcarcel**

Spain

**Crhistinne Cavalheiro Gonçalves**

Brazil

**Fidel Gonzalez**

USA

**Kevin Gorey**

Canada

**George Gotsadze**

Georgia

**Kaine Grigg**

Australia

**John Grundy**

Australia

**Danan Gu**

USA

**Ming Guan**

China

**Per Gustafsson**

Sweden

**Sally Guttmacher**

USA

**Clement Gwede**

USA

**Gerda-Maria Haas**

Germany

**Geraldine Pierre Haile**

USA

**Rita Hamad**

USA

**Wim Hardyns**

Belgium

**Patrick Harris**

Australia

**Lisa Harryson**

Sweden

**Tsovinar Harutyunyan**

Armenia

**Michael Hawkes**

Canada

**Lydia Hearn**

Australia

**Kristian Heggebø**

Norway

**Margaret Henning**

USA

**Julie Hepworth**

Australia

**Ileana Heredia-Pi**

Mexico

**Alison Hernández**

Sweden

**Peter Hill**

Australia

**Marion Hitzeroth**

Germany

**Ayako Hiyoshi**

Sweden

**Neil A Holtzman**

USA

**Monjurul Hoque**

South Africa

**Moshe Hosen**

Israel

**Ruwei Hu**

China

**Jia Hu**

USA

**Chengli Huang**

China

**Yu-Tung Huang**

Taiwan

**Susan Hughes**

USA

**Claire Ibell**

Australia

**Lawrence Ikamari**

Kenya

**Enrico Ivaldi**

Italy

**Heiko J. Jahn**

Germany

**Urban Janlert**

Sweden

**Kari Jarvinen**

Australia

**Saroj Jayasinghe**

Sri Lanka

**Weiyan Jian**

China

**Yi Jiang**

China

**Pingyue Jin**

China

**William Joe**

India

**Hannah Jordan**

UK

**Clara Juárez-Ramírez**

Mexico

**Regina Jutz**

Germany

**Anuska Kalita**

India

**Demy Kamboukos**

USA

**Aaron Katz**

USA

**Amy Katz**

Canada

**Zahra Kavosi**

Iran

**Ernest Kenu**

Ghana

**Zeinab Khadr**

Egypt

**Ali S Khashan**

Ireland

**Wahida Kihal**

France

**Hak-Seon Kim**

Korea, Republic of

**Hongsoo Kim**

Korea, Republic of

**Jong In Kim**

Korea, Republic of

**Tae Hyun Kim**

Korea, Republic of

**Sylvia Kirchengast**

Austria

**Jenny Knight**

Australia

**Peter Kolarcik**

Slovakia

**Katarzyna Kolasa**

Poland

**Agnes Kotoh**

Ghana

**Steinar Krokstad**

Norway

**Gunnar Kullgren**

USA

**August Kuwawenaruwa**

Tanzania

**Evelyn Kwakye**

USA

**Brendan Kwesiga**

Uganda

**Dejian Lai**

USA

**K Robert Lai**

Taiwan

**Hui-Chu Lang**

Taiwan

**Nicholas Lardinois**

USA

**Mary Lashley**

USA

**Sergio Latorre-Arteaga**

Spain

**Jorgen Lauridsen**

Denmark

**Julian Le Grand**

UK

**Albert Lee**

Hong Kong

**Kyu Sik Lee**

Korea, South

**Jinhyung Lee**

Korea, Republic of

**Anja Leist**

Luxembourg

**Carolyn Lester**

UK

**Carey Levinton**

Canada

**Chunhui Li**

China

**Shu Qin Li**

Australia

**Hailun Liang**

USA

**Ying Liang**

China

**Seunghoo Lim**

Japan

**Kaibiao Lin**

China

**Martin Lindstrom**

Sweden

**Hong Liu**

China

**Wen Liu**

China

**Xiaoting Liu**

China

**Vincent Lorant**

Belgium

**Peiyi Lu**

China

**Liming Lu**

China

**Tsung-Hsueh Lu**

Taiwan

**Mzwandile Mabhala**

UK

**Jane Macha**

Tanzania

**Beatriz Macias**

Colombia

**James Macinko**

USA

**Laís Mais**

Brazil

**L Mantini**

Canada

**Abubakar Manu**

Ghana

**Paul Marschall**

Germany

**Felix Masiye**

Zambia

**Annette Maxwell**

USA

**Susanne Mayer**

Austria

**Maureen Mayhew**

Canada

**Maurice Mbago**

Tanzania

**Elizabeth Mcdonald**

Australia

**Joan Mcdowell**

UK

**John Mckie**

Australia

**Graham Meadows**

Australia

**Maria Gabriella Melchiorre**

Italy

**Andrew Millard**

UK

**Samuel Mills**

USA

**Kaelan Moat**

Canada

**Susanne Moebus**

Germany

**Katia Mohindra**

Canada

**Ali Montazeri**

Iran

**Camila Monteiro**

Brazil

**Stephanie Montesanti**

Canada

**Max Moreno Madriñán**

USA

**Muluneh Mossie**

Ethiopia

**Ani Movsisyan**

UK

**Oscar J Mujica**

USA

**Fatima Mukhtar**

Pakistan

**Moses Muriithi**

Kenya

**Namuunda Mutombo**

Kenya

**Nancy Muturi**

USA

**Adamson Sinjani Muula**

Malawi

**Adamson Muula**

Malawi

**Eun Woo Nam**

Korea, Republic of

**Agnes Nanyonjo**

Sweden

**Solange Nappo**

Brazil

**Linda Neuhauser**

USA

**Nobubelo Ngandu**

South Africa

**Nathan Nickel**

Canada

**Stephen Nkansah-Amankra**

USA

**Thomas Novotny**

USA

**Behdin Nowrouzi**

Canada

**Roberto Nuño-Solinis**

Spain

**Rozina Nuruddin**

Pakistan

**Walter Obiero**

USA

**Thomas O'connell**

Switzerland

**Fidelia Ohemeng**

Ghana

**Chinyere Okafor**

Nigeria

**Jerry Okal**

Kenya

**Joseph Okeibunor**

Congo

**John Oldroyd**

Australia

**Kristine Onarheim**

Norway

**Obinna Onwujekwe**

Nigeria

**Nkechi Onyeneho**

Nigeria

**Moe Ko Oo**

Thailand

**Arash Ordookhani**

USA

**Takashi Oshio**

Japan

**Marie-Jo Ouimet**

Canada

**Cesar Augusto Oviedo Tejada**

Brazil

**Cynthia Owsley**

USA

**Maharouf Oyolola**

Kenya

**Boniface Oyugi**

Kenya

**Manoranjan Pal**

India

**Bibhudutta Panda**

USA

**Supasit Pannarunothai**

Thailand

**Sekar Paramita**

Japan

**Ju Park**

Korea, Republic of

**Pavitra Paul**

Finland

**Anna Pearce**

UK

**Jean-François Pelletier**

Canada

**Sathira Perera**

Sri Lanka

**Will Perez**

Nicaragua

**Karen Pesse**

Ecuador

**Jane Phiri**

South Africa

**Rup Phukan**

India

**Jaime Pinilla**

Spain

**Mauricio Polidoro**

Brazil

**Susan Povall**

UK

**Ivan Pristas**

Croatia

**Steven Prus**

Canada

**Dongfu Qian**

China

**Rosanna Quattrin**

Italy

**Amelie Quesnel-Vallee**

Canada

**Luis Rajmil**

Spain

**Maria Rameka**

New Zealand

**Jose M Ramos**

Spain

**Vivian R Ramsden**

Canada

**Santosh Rath**

UK

**Daniel Reidpath**

Malaysia

**Rachel Reilly**

Australia

**Joerg Richter**

Norway

**Jan Rigby**

Ireland

**Assistant Prof Roberts**

USA

**Laura Rosella**

Canada

**Kevin Rowley**

Australia

**Ana Lorena Ruano**

Norway

**Mark Rubin**

Australia

**Asma Sabermahani**

Iran

**Marc Saez**

Spain

**Ceyda Sahan**

Turkey

**Mariano Salazar**

Sweden

**Carolina Sales**

Brazil

**Maria Sanchez**

Sweden

**Paula Santana**

Portugal

**Vilma Santana**

Brazil

**Narendra Nath Sarkar**

India

**Franziska Satzinger**

Germany

**Carme Saurina**

Spain

**Erik Schokkaert**

Belgium

**Lisa Scholin**

UK

**Julia Schröders**

Sweden

**Lars Schwettmann**

Germany

**Edson Servan-Mori**

Mexico

**Maninder Singh Setia**

India

**Efrat Shadmi**

Israel

**Hosung Shin**

Korea, Republic of

**Lei Si**

China

**Florence Sibeudu**

Nigeria

**Arjumand Siddiqi**

Canada

**Juan Simó-Miñana**

Spain

**Zeynep Simsek**

Turkey

**Abhinav Singh**

India

**Douglas Singh**

USA

**Adam Sirois**

USA

**Linda Slack-Smith**

Australia

**Joyce Slater**

Canada

**Mariana Socal**

USA

**Rene Soria-Saucedo**

Bolivia

**Kyriakos Souliotis**

Greece

**Leonard Sowah**

USA

**Ireneous N. Soyiri**

Malaysia

**Stuart Speedie**

USA

**Einav Srulovici**

Israel

**Maisha Standifer**

USA

**Gregory Stevens**

USA

**Matheus Stivali**

Brazil

**Alexandrina Stoyanova**

Spain

**Qiang Sun**

China

**Canlan Sun**

USA

**Yang Sun**

China

**Suparmi Suparmi**

Indonesia

**Jean Tafforeau**

Belgium

**Simbarashe Takuva**

South Africa

**Kk Tang**

Australia

**Shinichi Tanihara**

Japan

**Xiaojun Tao**

China

**Wassim Tarraf**

USA

**Francis Tayie**

USA

**Fabrizio Tediosi**

Switzerland

**Tomas Tefera**

Ethiopia

**Tiew-Hwa Teng**

Australia

**Angela Testi**

Italy

**Abha Tewari**

India

**Christine Therrien**

USA

**Engelbert Theurl**

Austria

**Heike Thiel De Bocanegra**

USA

**Susan Thomas**

Australia

**Margaret Thorogood**

UK

**Christiana Titaley**

Indonesia

**Claudia Travassos**

Brazil

**Vladimir Trkulja**

Croatia

**Jandryle Trondillo**

Philippines

**Cleon Tsimbos**

Greece

**Reginald Tucker-Seeley**

USA

**Geir Conrad Tufte**

Norway

**Peter Tugwell**

Canada

**Ho-Jui Tung**

Taiwan

**Peter Twum**

China

**Nkolika Uguru**

Nigeria

**Rosa M. Urbanos-Garrido**

Spain

**Lalitha Vadrevu**

India

**Masoud Vaezghasemi**

Sweden

**Nicole Valentine**

Switzerland

**Kjetil A. Van Der Wel**

Norway

**Ronan Van Rossem**

Belgium

**Christophe Vanroelen**

Belgium

**Felipe Vasquez Lavin**

Chile

**Gerry Veenstra**

Canada

**Venetia-Sofia Velonaki**

Switzerland

**Petra Verdonk**

Netherlands

**Michel Vernay**

France

**Georgia Verropoulou**

Greece

**Paolo Villari**

Italy

**Pekka Virtanen**

Finland

**Olaf Von Dem Knesebeck**

Germany

**Athanassios Vozikis**

Greece

**David Walsh**

UK

**Stephen Ojiambo Wandera**

Uganda

**Lidan Wang**

China

**Wenhua Wang**

China

**Peigang Wang**

China

**Xin Wang**

China

**Joseph Wang'ombe**

Kenya

**Kelly Watt**

Australia

**Jacqui Webster**

Australia

**Stefan Weinmann**

USA

**Ronald Wesonga**

Uganda

**Amanda Whittal**

Germany

**Aung Win**

USA

**Zhuochun Wu**

China

**Hongwei Xu**

USA

**Elsie Yan**

Hong Kong

**Tingzhong Yang**

China

**Qiang Yao**

China

**Mahmut Yardim**

Turkey

**Fang Ye**

China

**Chia-Feng Yen**

Taiwan

**Elias Yesuf**

Ethiopia

**Bin Yu**

China

**Alexandra Zabinski**

USA

**Leah Zallman**

USA

**Yi Zeng**

USA

**Xiaojuan Zhang**

China

**Huafeng Zhang**

Norway

**Yuejen Zhao**

Australia

**Chengchao Zhou**

China

**Cheryl Zlotnick**

Israel

